# The Validation of the SOMNOwatch™ Actigraphy System for Periodic Limb Movements in Sleep Assessment

**DOI:** 10.3390/clockssleep6040038

**Published:** 2024-10-10

**Authors:** Ekaterina Spektor, Ingo Fietze, Mikhail G. Poluektov

**Affiliations:** 1 Department of Neurology and Neurosurgery, I.M. Sechenov First Moscow State Medical University (Sechenov University), 119435 Moscow, Russia; rectorat@staff.sechenov.ru; 2 Somnico, 10117 Berlin, Germany

**Keywords:** periodic limb movements, polysomnography, actigraphy, validation, SOMNOwatch

## Abstract

There is a growing body of evidence regarding the clinical significance of PLMS, and this movement disorder is of concern in both clinical and scientific contexts. Leg actigraphy is a convenient and promising method for screening periodic limb movements in sleep (PLMSs). This study aims to demonstrate the reliability of the SOMNOwatch™ actigraph for detecting periodic limb movements in sleep. Twenty-eight patients, referred to a sleep laboratory for various sleep problems, underwent nocturnal polysomnography with simultaneous one-sided actigraphy using the SOMNOwatch™ actigraph. Recordings of leg movements obtained from both methods were manually scored, calculating the periodic limb movement index (PLMI). The agreement between the methods was assessed through correlation analysis and event-by-event comparison. The correlation between the PLMI derived from PSG and SOMNOwatch™ was high and statistically significant (r = 0.98, *p* < 0.0001). The SOMNOwatch™ demonstrated a sensitivity of 86.7% and a specificity of 92.3% in detecting PLMS. Similarly, for detecting patients with a PLMI equal to or greater than 15, the sensitivity was 85.7%, and the specificity was 95.2%.

## 1. Introduction

### 1.1. Background

Periodic limb movement in sleep (PLMS) is a sleep-related movement disorder characterized by repetitive episodes of stereotyped movements. These movements most frequently affect the lower extremities, typically with the involvement of big toe extension, often in combination with the partial flexion of the ankle, the knee, and, sometimes, the hip. When PLMS leads to clinical sleep disturbance or fatigue which cannot be attributed to another primary sleep disorder or etiology, it is classified as periodic limb movement disorder (PLMD) [1]. Most patients with PLMS are unaware of the condition, which, in this case, is considered a polysomnographic finding [2]. However, the clinical significance of PLMSs that do not meet the criteria for PLMD is disputable [2,3]. Although PLMS may contribute to frequent sleep disruption, it does not always result in non-restorative sleep [4,5,6] or excessive daytime sleepiness [7]. PLMS is also presumed to be associated with the change in autonomic nervous system tone and the corresponding cardiovascular effects, such as alterations in heart rate variability [8,9], followed by increases in heart rate [9] and blood pressure [10,11]. The repetitive changes in the autonomic tone might exacerbate the course of cardiovascular diseases, potentially increasing the risk of cardiovascular events. Despite these associations, the role of PLMS as a predictor of worse outcomes in patients with cardiovascular pathology remains underexplored.

However, studying PLMS requires performing nocturnal polysomnography (PSG), which is the “gold standard” for sleep studies. The diagnostic criteria for PLMS are determined for PSG and include four and more movements in each series with the duration of a single movement equal to or exceeding 0.5 s and less than 10 s and the duration of an inter-movement interval equal to or exceeding 5 s and less than 90 s [12,13]. The procedure of PSG is quite complicated and may seem to be excessive when solely focused on identifying the presence of periodic limb movements. In light of this, the utilization of leg actigraphy presents itself as a beneficial and promising alternative. Other advantages of actigraphy are its cost-effectiveness and convenience for patients because the usage of this method requires minimal preparation and does not entail any discomfort, as opposed to PSG. In addition, PSG is a quite expensive procedure, and the current trend leans towards finding more cost-efficient alternatives for assessing sleep parameters [14].

### 1.2. Related Works 

The evaluation of leg actigraphy as a diagnostic tool for quantifying PLMS has undergone systematic assessment by D. Plante [15]. While a limited number of large-scale studies have demonstrated the utility of lower-limb actigraphy in measuring periodic limb movements, there exists a considerable diversity in the devices used, the placement of the actigraph on the lower extremity, and the methods applied for quantifying PLMS. The sensitivity and specificity of these devices also vary, with potential differences linked to the technical characteristics of each device.

Validation studies for PLM detection, as reported in the peer-reviewed literature, have been conducted on the Cambridge Actiwatch AW-64 actigraph [16] and the PAM-RL actigraph [16,17]. In a study by E. Sforza et al., the sensitivity and specificity of the PAM-RL actigraph were reported to be 88% and 76%, respectively, with a correlation coefficient of 0.87 between the periodic limb movement index (PLMI) obtained by PSG and actigraphy [17]. Additionally, V. Gschliesser et al. found that the PLMI values derived from PAM-RL were significantly higher than those obtained from PSG, although the correlation between PAM-RL and PSG was high (rho = 0.939). The authors also demonstrated that the Actiwatch actigraph tends to underestimate the PLMI, correlating significantly with PSG but to a lesser extent than PAM-RL (rho = 0.835).

The Actiwatch actigraph’s capability to quantify PLMS has also been evaluated with the device positioned at the base of the big toe, as documented by Kemlink et al. [18]. The authors observed a significant correlation across all resulting parameters when comparing actigraphy versus PSG. Additionally, the actigraph placed at the toe exhibited a higher sensitivity (94% versus 67%), albeit a slightly lower specificity (91% versus 95%), compared to the ankle placement. 

The SOMNOwatchTM actigraph was examined with the following publication in peer-reviewed sources for distinguishing sleep from wakefulness only [19]. In this study, the device demonstrated a sensitivity of 90.2% and a specificity of 95.2%. Additionally, a small study by H. Benes et al. [20], published as an abstract, explored the utility of the SOMNOwatch™ actigraph in detecting PLMS. It described 19 patients with simultaneous PSG and actigraphy recordings and showed a sufficient agreement of leg activity measured by the SOMNOwatch with the EMG measures from polysomnography and a high degree of correlation between both measurements (r = 0.962). However, the algorithm used in the SOMNOwatchTM is the same as in PAM-RL, which has already been validated for quantifying PLM.

One of the notable limitations of the actigraphic method is its incapacity to integrate data from both legs [15]. This limitation can result in an overestimation of the PLMI when the total PLMS is calculated as a sum of the PLMS derived from each leg separately. In PSG records, simultaneous motion in both legs is typically treated as a single movement, whereas actigraphy might interpret the same data as distinct movements. The approach to PLMS estimation in validation studies varies, with some comparing data from one leg to the corresponding EMG channel [21], while others compare the total PLMS obtained from both limbs with the total PLMS in PSG records [16,17]. Each method has its strengths and limitations: the former provides more precise information about the actigraph’s ability to detect single motions, while the latter allows for assessing the device’s utility in diagnosing PLMS in clinical settings.

The SOMNOwatch^TM^ (SOMNOmedics, Randersacker, GmbH) is a compact and waterproof motion detection system that concurrently records acceleration in the x, y, and z directions, along with the patient’s position and ambient light levels. We hypothesize that the SOMNOwatch™ actigraph provides a reliable and non-inferior alternative to nocturnal polysomnography in detecting periodic limb movements during sleep. This study aims to validate this hypothesis by directly comparing the performance of the SOMNOwatch™ with the established gold standard for diagnosing sleep movement disorders.

## 2. Results

A total of 28 people (14 of them male, 50%) were included in this study. The median age was 42.5 years, the interquartile range was 33.25–61.75 years, and the absolute range was 20–82 years. Diagnoses were established after the clinical interview, examination, and PSG, revealing obstructive sleep apnea syndrome (OSAS) in 11 patients (39.3%), restless legs syndrome in 7 patients (25%), insomnia disorder in 3 patients (10.7%), snoring in 3 patients (10.7%), parasomnia in 2 patients (7.1%), panic attack in 1 patient (3.6%), and sleep myoclonus in 1 patient (3.6%).

The majority of included patients had obstructive sleep apnea syndrome or restless legs syndrome, which appears reasonable because these disturbances are accompanied by an increased periodic limb movement index. Overall, a large range of PLMIs was presented in the studied sample. The values of the PLMI in the different nosology subgroups are presented in Table 1. The mean PLMI obtained by polysomnography was equal to 13.8/h (±24.2/h), and the same variable for the actimetry measurement was equal to 12.8/h (±20.9/h).

**Figure 1:**
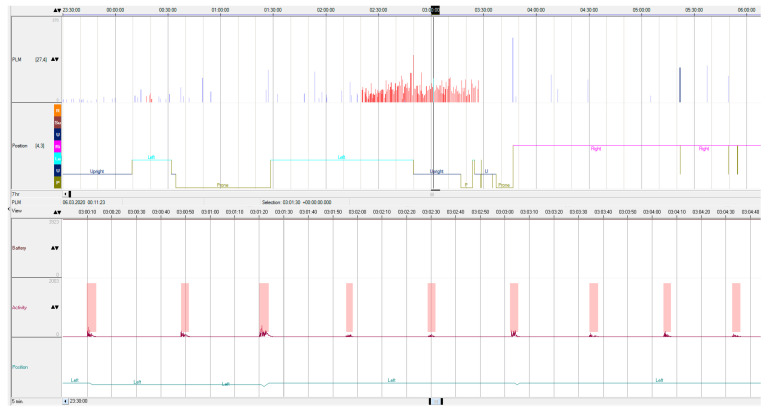
Actigraphic raw data. The lower section of the window depicts a 5 min epoch with a series of periodic limb movements, while the upper section of the window displays movements throughout the entire night, with PLMS marked in red.

**Figure 2:**
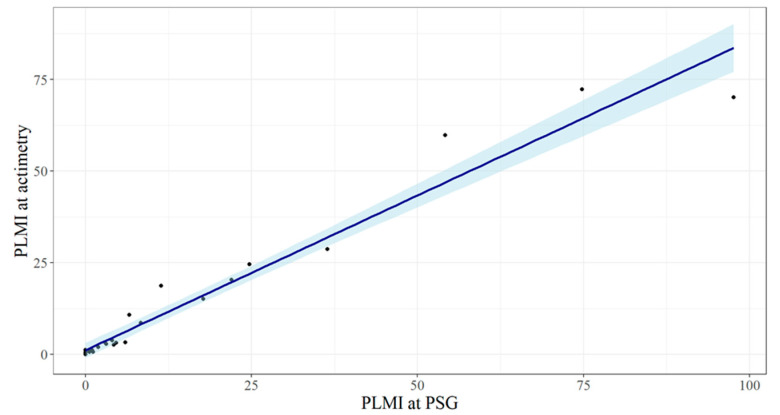
Scatter plot for the measurements of the periodic limb movement index (PLMI) by polysomnography (PSG) and actimetry.

The comparison of the PLMI obtained by the two methods using the Wilcoxon signed rank test for paired data shows no statistically significant differences (*p* = 0.33). To assess the accordance of the PLMI measured by “gold standard” polysomnography and actimetry with the SOMNOwatch^TM^, the Pearson correlation coefficient was calculated, and a Bland–Altman plot was constructed. Figure 1 illustrates an example of the actigraphic recording obtained during the nocturnal study. The indexes of the periodic limb movements derived by the two methods demonstrated a highly linear correlated relationship (Figure 2), with Pearson’s r equal to 0.98, 95% CI 0.95–0.99 (*p* < 0.0001).

**Figure 3:**
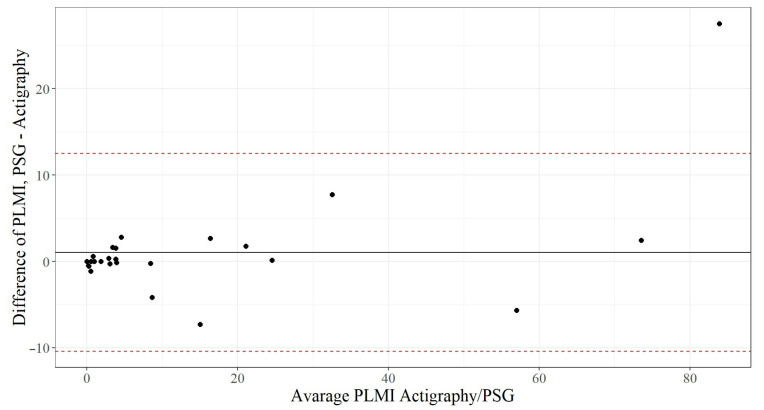
Bland–Altman plot: average PLMI measured via actigraphy and PSG versus the difference in the PLMI of both methods. PLMI—periodic limb movement index; and PSG—polysomnography.

The Bland–Altman plot (Figure 3) demonstrates that the PLMI measured by polysomnography is, on average, 1.05 ± 5.8 movements higher than the PLMI measured via actigraphy. The dotted red lines represent ±1.96 standard deviation, and 27 of 28 observations (96.4%) are within this scope. The differences are likely to be normally distributed, and, therefore, it could be concluded that 95% of all measurement differences for the PLMI lie between the agreement lines of −10.3 and +12.4. The graph also shows that the more PLMI a patient had, the more difference between the two methods.

An event-by-event comparison was conducted to calculate the sensitivity, specificity, and accuracy of the SOMNOwatch^TM^ in detecting individual periodic limb movements. A true-positive result was defined as the number of movements that met the PLMS criteria and were simultaneously registered by both PSG and actigraphy. A false-positive result was defined as the number of movements that met the PLMS criteria and were detected by the SOMNOwatch™ but not by PSG. A false-negative result was defined as the number of movements that met the criteria of PLMS but were missed by the SOMNOwatch™ and were detected by PSG only. Lastly, a true negative result was defined as the number of inter-movement intervals free of PLMS that matched on both recordings. Each of these parameters was derived from the entire sample and is listed in the contingency matrix (Table 2).

Based on the derived values, the accuracy (1), sensitivity (2), and specificity (3) of the SOMNOwatch^TM^ were calculated and were 89.5%, 86.7%, and 92.3%, respectively.

(1) $$\mathrm{Accuracy}=\frac{TP+TN}{TP+FP+TN+FN}=\frac{4314}{4821}=0.895$$

(2) $$\mathrm{Sensitivity}=\frac{TP}{TP+FN}=\frac{2080}{2400}=0.867$$

(3) $$\mathrm{Specificity}=\frac{TN}{FP+TN}=\frac{2234}{2421}=0.923$$

The evaluation of the SOMNOwatch™ to detect an abnormal level of PLMS, defined as equal to or exceeding 15 movements per hour of sleep, was conducted separately, and a contingency matrix was also constructed (Table 3). 

Thus, the accuracy (4), sensitivity (5), and specificity (6) of the SOMNOwatch^TM^ in detecting an increased PLMI were 92.9%, 85.7%, and 95.2%, respectively.

(4) $$\mathrm{Accuracy}=\frac{TP+TN}{TP+FP+TN+FN}=\frac{26}{28}=0.929$$

(5) $$\mathrm{Sensitivity}=\frac{TP}{TP+FN}=\frac{6}{7}=0.857$$

(6) $$\mathrm{Specificity}=\frac{TN}{FP+TN}=\frac{20}{21}=0.952$$

## 3. Discussion

The results obtained in the current study demonstrate that the SOMNOwatch™ actigraph is a reliable and convenient tool for quantifying PLMS and detecting pathological PLMI. A notable strength of this research lies in the heterogeneous sample of patients with various sleep problems, resulting in a diverse range of leg movements during sleep. Despite the variability in sleep problems within the studied sample, disorders associated with an increased PLMI (such as OSAS and RLS) were predominant. Consequently, the range of PLMIs in the sample was sufficiently broad, encompassing both the absence of PLMS and very high PLMS indexes. This diversity allows us to assess the device’s performance accurately in different clinical situations.

The findings confirm that the SOMNOwatch™ exhibits high specificity and sensitivity for both quantifying PLMS and determining the presence of a pathological PLM index, defined as 15 or more movements per hour of sleep. This study represents the first validation of the SOMNOwatch™ specifically for the detection of PLMS, addressing a critical gap in the current literature. By establishing the SOMNOwatch™ as a reliable tool for PLMS assessment, our findings provide a valuable resource for other researchers and clinicians seeking to study or diagnose sleep-related movement disorders using this device.

In comparison with PAM-RL and Actiwatch devices, the SOMNOwatch™ demonstrated superior accuracy, sensitivity, and specificity. It is noteworthy, however, that the PLMSs in past PAM-RL and Actiwatch studies were automatically scored and calculated. In our current research, manual scoring was employed for two reasons. First, we believe that adhering to established standards for scoring leg movements is essential, even if these standards were initially developed for polysomnography. Second, manual scoring provides more precise and authentic information about leg movements. An exploratory analysis revealed the low quality of automatic processing and the necessity for manual correction. Although this approach makes the actigraphic method less convenient in clinical settings due to the time spent on data processing, the results obtained in this manner reflect the actual PLMI. Nevertheless, the sensitivity and specificity values obtained in this study cannot be directly compared with the corresponding values of other actigraphic systems. This is the first limitation of our study. 

Another limitation of this study is the one-sided registration of leg movements. In clinical settings, the usual objective is to assess movements from both legs, resulting in the PLM index as an integral value. Existing evidence indicates that the actigraphic method tends to overestimate the PLMI because it calculates indexes from two devices placed on two ankles instead of combining and assessing them together, similar to a polysomnography approach. Of practical interest is understanding the degree of this overestimation. However, it is important to note that the extent of overestimation does not depend on the device’s quality but on the percentage of bilateral movements, which is a somewhat random factor. If most PLMSs during the night are bilateral, the overestimation would be high because all these movements will be summarized. Conversely, if a patient has unilateral movements only, the concordance between PSG and actigraphy will be as high as the quality that the actigraphic system allows. Therefore, considering the influence of this random factor that does not depend on the actigraph’s quality, the more crucial issue is determining the device’s ability to detect single movements. For this purpose, the one-sided mode of registration appears to be more suitable, especially when manual scoring is applied.

In the current research, the Pearson correlation coefficient obtained is 0.98, and the total accuracy is estimated at 89.5%. In another study of the SOMNOwatch™, the correlation (r) was reported as 0.962, and the accuracy ranged between 91% and 99% [20]. Our results demonstrate a higher correlation but a slightly lower accuracy of the device. It is important to note that the design of the research conducted by Benes et al. differs from our current study. Firstly, the authors assessed both PLMS and PLMW, and, secondly, recordings with the actigraph were derived from both legs. However, despite these minor differences, both studies consistently showed results for predicting the PLMI. The high values of accuracy and correlation with the PLMI measured by PSG indicate the utility of the SOMNOwatch™ in clinical practice. On the other hand, the usage of the SOMNOwatch™ for PLMD diagnosis is limited due to its inability to identify periodic limb movements with arousals and, therefore, calculate the periodic limb movements with arousals index (PLMAI). This index is relevant for a possible treatment decision. Nevertheless, a high PLMI obtained by the SOMNOwatch™ together with sleepiness or non-restorative sleep could be an indication for a PSG.

## 4. Materials and Methods

### 4.1. Participants

The sample size was calculated according to Formula (7), proposed by N. A. Charoo et al. [22]. Reliability and confidence were equal to 90% and 95%, respectively.

(7) $$n=\frac{\ln\;(1-Confidence)}{\ln\;(Reliability)}=\frac{\ln\;(1-0.95)}{\ln\;(0.90)}=28$$

The study group comprised a heterogeneous adult population referred to a sleep laboratory for various reasons (insomnia, snoring, RLS, parasomnias). A total of 28 participants were included (14 men, 14 women, mean age 47.9 ± 18.1 years, range 20–82). The leg movements of the patients were recorded simultaneously for one night using polysomnography electromyography (EMG) and right-leg actigraphy. The details of the leg movement assessments are described below. All patients were informed about the purpose of this study and gave written informed consent.

### 4.2. Polysomnography

All participants underwent nocturnal stationary polysomnography (SOMNOscreen™, SOMNOmedics GmbH, Randersacker, Germany). The polysomnography was performed using the following montage: electroencephalography (EEG, recordings Fp1A2, Fp2A1, C3A2, C4A1, O1A2, O2A1), two electrooculography (EOG) channels, submental EMG, respiratory flow signal, registration of chest and abdominal movements, two EMG channels for right and left tibialis anterior muscles, electrocardiography (ECG), arterial oxygen saturation (SaO2), registration of body position, and video recording. The quality of the EMG recording was verified by asking the patient to bend their feet. Sleep scoring was performed manually according the criteria of The American Association of Sleep Medicine Manual for the scoring the sleep and associated events, version 2.6 (2020) [23]. The processing of records was performed with the software “DOMINO^®^ version 2.9.0” (SOMNOmedics GmbH, Randersacker, Germany).

Leg movements were calculated in accordance with the rules for recording and scoring periodic limb movements in sleep and wakefulness determined by the International Restless Legs Syndrome Study Group in 2006 [24] and then revised in 2016 [12]. Other polysomnographic parameters that are not relevant to the study aim were also obtained, but they are not considered here.

The total number of periodic limb movements (PLMSs) and the PLMI were calculated. Movements with a duration equal to or exceeding 0.5 s were regarded as leg movements during sleep (LMs) and those with a duration of less than 10 s as candidate LMs [12]. Two to four unilateral candidate LMs from two legs overlapping one another within a 0.5 s interval were considered a bilateral candidate LM if the total duration was less than 15 s. PLMSs were defined as candidate LMs if they comprised series of 4 or more, separated by more than 5 and less than 90 s and not interrupted by LM > 10 s. The PLMI was calculated as the number of PLMSs per hour of sleep [12].

### 4.3. Actigraphy

All participants underwent simultaneous overnight one-sided leg actigraphy alongside nocturnal polysomnography. Leg movements were recorded using a SOMNOwatchTM recorder placed on the patient’s right ankle. This device, based on accelerometry, can detect limb movements and differentiate between standing and lying using the integrated position sensor. Derived raw data were manually analyzed using the SOMNOwatch software “DOMINO^®^ Light 1.4” (SOMNOmedics GmbH, Randersacker, Germany) to determine the leg movements.

The actigraphic data underwent processing by calculating the same values as those for PSG: the total number of periodic limb movements and the number of PLMSs per hour of sleep (PLMI). A trained somnologist manually scored the PLMSs during sleep. Movements attributed to changes in body position and those with durations less than 0.5 s or exceeding 10 s were not considered as candidate LMs. PLMSs were defined as LM series comprising 4 or more movements, separated by more than 5 and less than 90 s, and not interrupted by LMs exceeding 10 s. The PLMI was then calculated as the number of PLMSs per hour of sleep [12].

### 4.4. Statistical Analysis

A statistical analysis was performed using R Statistical Software (version 4.1.3, R Foundation for Statistical Computing, Vienna, Austria). The descriptive statistics were reported as a median and interquartile range due to the small number of observations.

The primary outcome of the study was the PLMI derived from right-channel electromyography of PSG (PSG PLMI), which was regarded as the “gold standard”, and the PLMI derived from an actigraph placed on the right ankle, which was the analyzed measure. The secondary outcome was the presence of a PLMI above 15 movements per hour as a nominative variable. This is due to a fact that a PLMI above 15 is one of the diagnostic criteria of periodic limb movement disorder. Understanding whether actigraphy is sensitive enough to detect an abnormal PLMI is crucial in assessing its diagnostic capabilities.

To assess the correspondence between measurements obtained through actigraphy and PSG, a Spearman correlation analysis was conducted. PLM indexes obtained through both methods were compared using the Wilcoxon signed rank test for paired data, with a significance level set at 0.05. Describing the agreement between the two measurements, a Bland–Altman plot was developed. Event-by-event comparison was performed to evaluate sensitivity (the effectiveness of actigraphy in detecting PLMSs), specificity (the effectiveness in detecting the absence of movement or non-periodic movement), and overall accuracy or agreement. The signal detected simultaneously by both measurement devices was considered a true positive. A signal recorded during electromyographic monitoring but not detected by the actigraph was considered a false negative. When limb movement was detected by the actigraph without corresponding changes in the electromyogram, the signal was considered a false positive. The absence of limb movements in both methods was regarded as a true negative result. Sensitivity, specificity, and accuracy were also calculated, specifically focusing on actigraphy’s ability to identify an elevated PLMI, defined as a PLMI above 15 movements per hour of sleep.

## 5. Conclusions

Periodic limb movements in sleep, as measured with the SOMNOwatch™, demonstrated agreement with nocturnal polysomnography, which remains the “gold standard” for sleep studies. In this study, the sensitivity and specificity of PLMS detection were 86.7% and 92.3%, respectively, while the sensitivity and specificity for detecting a PLMI above 15 movements per hour (clinically significant rate of PLMSs) were 85.7% and 95.2%. It can be concluded that the SOMNOwatch™ is a reliable and accurate device for detecting periodic limb movements and may be utilized in clinical practice for PLMS screening in the general population.

## Figures and Tables

**Table 1:** PLMI values in the different nosology subgroups. RLS—restless legs syndrome; OSAS—obstructive sleep apnea syndrome; PSG PLMI—periodic limb movement index by polysomnography; ACT PLMI—periodic limb movement index by actigraphy; and Me (Q1~Q3)—median and interquartile range.

Nosology	PSG PLMI, Me (Q1~Q3)	ACT PLMI, Me (Q1~Q3)
OSAS	4.25 (1.59~7.92)	3.6 (1.62~10.22)
RLS	22 (5.7~45.3)	20.25 (9.63~44.27)
Insomnia disorder	3.12 (1.56~3.56)	2.75 (1.63~3.23)
Others	1.45 (0.43~4.7)	1.45 (0.43~2.76)

**Table 2:** Contingency matrix for PLMS predicted by actigraphy. PLM—periodic limb movement; PSG—polysomnography; TP—true positive; FP—false positive; TN—true negative; and FN—false negative.

	Presence of PLM by Actimetry	Absence of PLM by Actimetry	Sum
Presence of PLM by PSG	2080 (TP)	320 (FN)	2400
Absence of PLM by PSG	187 (FP)	2234 (TN)	2421
Sum	2267	2554	4821

**Table 3:** Contingency matrix for PLMS equal to or above 15/h predicted by actigraphy.

	PLMI ≥ 15 by Actimetry	PLMI < 15 by Actimetry	Sum
PLMI ≥ 15 by PSG	6 (TP)	1 (FN)	7
PLMI < 15 by PSG	1 (FP)	20 (TN)	21
Sum	7	21	28

## Data Availability

The data supporting the reported results, including the anonymized demographic and clinical data of patients and the results from nocturnal polysomnography and actigraphy measurements, are available in the Supplementary Materials of this article. The dataset is provided as an .xlsx file and can be accessed and downloaded directly from the journal’s Supplementary Materials section.

## Supplementary Materials

The supporting information about participants can be downloaded at https://www.mdpi.com/article/10.3390/clockssleep6040038/s1.
